# Interactions of zinc- and redox-signaling pathways

**DOI:** 10.1016/j.redox.2021.101916

**Published:** 2021-02-24

**Authors:** Christopher Hübner, Hajo Haase

**Affiliations:** Technische Universität Berlin, Chair of Food Chemistry and Toxicology, Straße des 17. Juni 135, 10623, Berlin, Germany

**Keywords:** Zinc, ROS, Redox metabolism, Phosphatases, Caspases, Metallothionein

## Abstract

Zinc and cellular oxidants such as reactive oxygen species (ROS) each participate in a multitude of physiological functions. There is considerable overlap between the affected events, including signal transduction. While there is no obvious direct connection between zinc and ROS, mainly because the bivalent cation zinc does not change its oxidation state in biological systems, these are linked by their interaction with sulfur, forming the remarkable triad of zinc, ROS, and protein thiols. First, zinc binds to reduced thiols and can be released upon oxidation. Thereby, redox signals are translated into changes in the free zinc concentration, which can act as zinc signals. Second, zinc affects oxidation of thiols in several ways, directly as well as indirectly. A protein incorporating many of these interactions is metallothionein (MT), which is rich in cysteine and capable of binding up to seven zinc ions in its fully reduced state. Zinc binding is diminished after (partial) oxidation, while thiols show increased reactivity in the absence of bound metal ions. Adding still more complexity, the MT promoter is controlled by zinc (via metal regulatory transcription factor 1 (MTF-1)) as well as redox (via nuclear factor erythroid 2-related factor 2 (NRF2)). Many signaling cascades that are important for cell proliferation or apoptosis contain protein thiols, acting as centers for crosstalk between zinc- and redox-signaling. A prominent example for shared molecular targets for zinc and ROS are active site cysteine thiols in protein tyrosine phosphatases (PTP), their activity being downregulated by oxidation as well as zinc binding. Because zinc binding also protects PTP thiols form irreversible oxidation, there is a multi-faceted reciprocal interaction, illustrating that zinc- and redox-signaling are intricately linked on multiple levels.

## Graphical abstract

**Figure:**
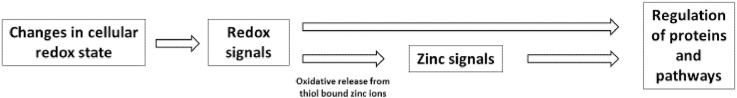

## Abbreviations

ZIP: Zrt-, Irt-like protein
ZnT: zinc transporter
MT: metallothionein
LPS: lipopolysaccharide
ROS: reactive oxygen species
RS: reactive species
RNS: reactive nitrogen species
NOS: NO synthase
SOD: superoxide dismutase
MAO: Monoamine oxidases
α-KDH: α-ketoglutarate dehydrogenase
NAD^+^: nicotinamide adenine dinucleotide
XO: xanthine oxidase
XDH: xanthine dehydrogenase
ER: endoplasmic reticulum
GSH: glutathione
GSSG: glutathione disulfide
MMO: microsomal monooxygenase
NADPH: nicotinamide adenine dinucleotide phosphate
Nox: NADPH oxidase
GCS: glutamate cysteine synthetase
T_red_: thionein
MTF-1: metal regulatory transcription factor 1
MRE: metal response element
HPLC: high performance liquid chromatography
SDS-PAGE: sodium dodecyl sulfate polyacrylamide gel electrophoresis
PTP: protein tyrosine phosphatase
PSP: protein serine/threonine phosphatase
DUSP: dual-specificity phosphatases
EDTA: ethylenediaminetetraacetic acid
TCEP: tris(2-carboxyethyl)phosphine
PTEN: phosphatase and tensin homolog deleted on chromosome 10
MAKP: MAPK phosphatase
ARE: antioxidant response element
GRE: glucocorticoid response element
Nrf2: nuclear factor erythroid 2-related factor 2
DNA: deoxyribonucleic acid
Keap1: Kelch-like ECH-associated protein 1
NF-κB: nuclear factor κB
MAPK: mitogenactivated protein kinase
AP-1: activator protein 1
PKC: protein kinase C
JNK: Jun N-terminal kinases
ERK: extracellular signal-regulated kinases
MEK: mitogen activated protein kinase kinase

## 1 Introduction

The trace element zinc is essential for all living organisms. Jules Raulin discovered already in 1869 that zinc is required for the growth of *aspergillus niger.* [1] Subsequently the biological relevance of zinc was recognized for an increasing number of organisms. E.g., in the 1930's zinc was first described to be essential for rats [2]. Finally, in the 1960's Ananda Prasad and colleagues reported its essentiality for humans [[2], [3], [4]]. On the molecular level, the observation that carbonic anhydrase contains zinc, which is required for enzymatic activity [5], instigated the identification of many further biochemical roles of zinc. In subsequent decades the recognition of zinc in biochemistry increased substantially, as comprehensively reviewed by Maret [6]. Today, many zinc-dependent physiological functions are known. For example, zinc has a crucial role in all phases of wound healing, which is impaired by dysregulated zinc homeostasis [7]. Moreover, the function of the immune system is strongly dependent on zinc. As a second messenger for immune cell signaling, and thus for the functionality of immune cells, zinc is an important regulator of the immune response [[8], [9], [10]].

Many biological processes are redox-dependent [11]. The electron transfer during these redox reactions may lead to the formation of reactive oxygen species (ROS), initializing further redox-processes [12,13]. For maintaining numerous physiological functions a balance between ROS production and ROS elimination is crucial. An overproduction of ROS or lack/inhibition of radical-depleting enzymes leads to dysregulation of this equilibrium and potentially to oxidative stress [14]. The latter is associated with the development of diabetes and cancer, as well as cardiovascular, neurodegenerative, and many other diseases [[15], [16], [17]]. To ensure an appropriate redox homeostasis many different factors are required, one of them being zinc. Zinc itself is redox-inactive and always present in the valence state Zn(II) in biological systems. Thus, zinc cannot directly participate in redox reactions. However, several of its ligands are susceptible to reduction or oxidation, resulting in zinc-binding or oxidative release, respectively. In turn, bound zinc influences the oxidation of its ligands, affecting, e.g., redox modulation of protein function [18,19]. Hence, there is a significant link between zinc and redox metabolism and the functions of zinc in redox metabolism and the crosstalk between zinc signaling and redox signaling will be summarized below.

## 2 Sources of ROS and important redox couples

Many biological processes under aerobic conditions involve the formation of ROS. These are highly reactive molecules that can function as cell signaling components or may act as toxic metabolites [20]. The term ROS comprises radical molecules (e.g., O_2_^•-^, HO^•^) or non-radical molecules, such as H_2_O_2_. ROS are produced at different intracellular sites and vary in their properties with regard to reactivity, half-life time and other factors. E.g., the half-life time of HO^•^ is over 1000 times lower than for H_2_O_2_. Accordingly, in contrast to H_2_O_2_, HO^•^ swiftly reacts very close to the site of its formation [21]. It has long been known that high concentrations of ROS can react with a range of cellular macromolecules, such as lipids, proteins or nucleic acids, inducing oxidative cell damage. Due to these characteristics they were often exclusively associated with pathophysiological processes. However, nowadays it is known that ROS have many physiological functions, as well, and adequate concentrations are essential, e.g., for signal transduction [[22], [23], [24], [25]]. In most cases, ROS, such as H_2_O_2_ or O_2_^•-^ are generated from molecular oxygen, thus ROS production is dependent on the oxygen concentration [[26], [27], [28]]. The oxygen partial pressure *in vivo* differs considerably between different tissues (1–100 mm Hg). However, the oxygen concentration mainly ranges between 1 – 10 mm Hg in cells *in vivo* and only in the lung or some arteries higher concentrations of up to 100 mm Hg occur [29,30]. Hence, the generated ROS concentrations and their potential to influence physiological processes, such as signal transduction, vary between different tissues and cells.

The physiological relevance of cell culture experiments is affected by the availability of oxygen, as well. Most of the experiments that will be discussed in this article were conducted under *in vitro* cell culture conditions. Typical cell culture conditions (95% air/5% CO_2_) correspond to an oxygen partial pressure of 150 mm Hg O_2_ [29], significantly exceeding the abovementioned oxygen partial pressure *in vivo* of 1 to 100 mm Hg [30]. In case of high O_2_ concentrations the ROS production is increased [26,27], resulting in significant differences of cellular ROS levels between *in vivo* and *in vitro* conditions. In addition, common cell culture media do not contain antioxidants, such as tocopherol or ascorbate, because under these conditions they are insoluble or instable, respectively. Furthermore, selenium, which is necessary for antioxidative systems, such as glutathione peroxidases or for the immune system, is often deficient in cell culture media [31,32]. Hence, *in vitro* higher amounts of ROS and lower availability antioxidants lead to prooxidative conditions and induction of signaling pathways, which would not be activated in a comparable fashion *in vivo* [33].

Similar to ROS there exist other reactive species (RS) of physiological relevance. These include reactive nitrogen species (RNS) or reactive sulfur species [34,35]. An example for the “Janus-faced functions” of RNS is nitric monoxide (NO^•^) [36]. The formation of NO^•^ from arginine is catalyzed by three isoforms of NO synthase (NOS): neuronal NOS, inducible NOS, and endothelial NOS. A detailed summary of the three isoforms and their different functions is provided by Förstermann et al. [37] On the one hand, NO^•^ is important for vasodilation of blood vessels [38]. Furthermore NO^•^ modulates signaling cascades, regulating the activity of guanylate cyclase and hereby the formation of the second messenger guanosine 3':5'-cyclic monophosphate [39]. On the other hand, NO^•^ can inhibit enzymes, such as catalase [40], or react with O_2_^•-^, resulting in the formation of peroxynitrite [41,42]. Comparable to NO^•^, peroxynitrite has beneficial as well as damaging effects. It is a strong oxidant able to kill pathogens [43], but may also lead to cell damage by the oxidation of membrane lipids or the inhibition of the mitochondrial electron transport chain [44,45]. In addition, spontaneous homolytic cleavage of peroxynitrite leads to generation of free radicals such as HO^•^ and ^•^NO_2_, which also induce cellular damage [46].

ROS can originate from different intracellular sources (Table 1). Due to their role in respiration, mitochondria contribute significantly to cellular ROS production [47,48]. The respiratory chain contains multiple sites where O_2_^•-^ is generated through the reduction of oxygen. It is assumed that complex I and complex III are the main sites of O_2_^•-^ production in the respiratory chain [49,50]. Both complexes release O_2_^•-^ into the mitochondrial matrix while complex III also releases O_2_^•-^ into the intermembrane space, because complex III includes two pools of ubiquinone, which are in contact with either matrix or intermembrane space [51]. Under certain conditions succinate dehydrogenase, also known as complex II, contributes significantly to mitochondrial ROS production and is located in the inner mitochondrial membrane, facing toward the mitochondrial matrix, wherein the O_2_^•-^ is released [52]. O_2_^•-^ is either converted into H_2_O_2_ by superoxide dismutase (SOD), a more stable secondary product or can react with other molecules such as NO^•^ within the mitochondrial matrix (Table 2) [50,[53], [54], [55]]. The generated H_2_O_2_ mainly moves into the cytosol, however, a part will be decomposed by enzymes such as catalase, peroxiredoxin or glutathione peroxidase in both cellular compartments [56,57]. In addition, H_2_O_2_ can participate in the Fenton reaction when free iron ions are present, causing formation of radicals, hereby inducing a prooxidative milieu in mitochondria and other cellular compartments (Table 2) [58]. In addition to the complexes in the respiratory chain, ROS are also generated by various oxidoreductases localized either within one of the mitochondrial membranes or in the mitochondrial matrix. Monoamine oxidases (MAO) as an integral enzyme of the outer membrane catalyze the degradation of biogenic amines with the formation of H_2_O_2_ as a side product. Hauptmann et al. have shown that the oxidation of tyramine catalyzed by MAO generates H_2_O_2_ [59]. The enzyme α-ketoglutarate dehydrogenase (α-KDH) is localized at the inner membrane. For its function in the citrate acid cycle α-KDH requires nicotinamide adenine dinucleotide (NAD^+^) as electron acceptor during the oxidative decarboxylation from α-ketoglutarate to succinyl-CoA. A low level of NAD^+^, and thus a high NADH/NAD^+^ ratio, can lead to H_2_O_2_ production by α-KDH [60]. More examples for oxidoreductases generating ROS in mitochondria were reviewed elsewhere [47,61].

**Table 1:** Selected sources of cellular ROS in mammalian metabolism.

Organelle	Generated ROS	Molecular source	References
Mitochondria	O_2_^•-^	Respiratory chain (complex I, II and III)	[[49], [50], [51], [52]]
	H_2_O_2_	Monoamino-oxidase	[59]
	H_2_O_2_	α-ketoglutarate dehydrogenase	[60]
	O_2_^•-^	Cytochrome *b5* reductase	[62]
	H_2_O_2_	Glycerol 3 phosphate dehydrogenase	[63]
	H_2_O_2_, O_2_^•-^	Dihydroorotate dehydrogenase	[64]
Peroxisomes	H_2_O_2_	Acyl-CoA oxidases	[65]
	H_2_O_2_, O_2_^•-^	Xanthine oxidase	
	H_2_O_2_	d-amino acid oxidase	
	H_2_O_2_	Pipecolic acid oxidase	
	H_2_O_2_	d-aspartate oxidase	
	H_2_O_2_	Sarcosine oxidase	
	H_2_O_2_	L-alpha-hydroxy acid oxidase	
	H_2_O_2_	Polyamine oxidase	
Cytosol and Plasma membrane	H_2_O_2_, O_2_^•-^	Xanthine oxidase	[66]
	H_2_O_2_, O_2_^•-^	NADPH-oxidase	[67,68]
Endoplasmic reticulum (ER)	H_2_O_2_	Oxidative protein folding	[69,70]
	O_2_^•-^	NADH cytochrome b5 reductase	[71]
	H_2_O_2_, O_2_^•-^	Microsomal monooxygenase	[72]
Lysosomes	H_2_O_2_, O_2_^•-^	Lysosomal membrane	[73,74]

**Table 2:** Selected consecutive reactions of ROS.

ROS	Consecutive reactions	References
O_2_^•-^	Reaction with NO^•^→ Formation of ONOO^-^Conversion into H_2_O_2_	[50,53,54]
H_2_O_2_	Decomposition by catalase or other enzymes Fenton reaction → Formation of OH^•^	[[55], [56], [57], [58]]
HO^•^	Reaction with biomolecules (proteins, lipids) Induction of DNA damage	[58,75]

Apart from mitochondria, ROS can also be generated by other cellular organelles. Peroxisomes contain many redox-enzymes for performing their physiological tasks, e.g., β-oxidation of fatty acids. Peroxisomal enzymes, in particular oxidases, produce considerable amounts of ROS [76]. The reactions responsible for formation of ROS are similar to the ones in mitochondria, which are described above. Relevant peroxisomal ROS are O_2_^•-^ and H_2_O_2_, whereby the latter may react with metal ions, especially iron, in a Fenton reaction to generate HO^•^ (Table 2) [77]. Peroxisomes also contain ROS-scavenging enzymes, such as catalase, peroxidases or dismutases to detoxify ROS [[78], [79], [80]]. In addition, inducible NOS-generated NO^•^ may lead to formation of peroxynitrite [81]. Another example for a peroxisomal oxidase is xanthine oxidase (XO) [65]. XO is also present in the cytosol and is one of two forms of xanthine oxidoreductases. The other form is the xanthine dehydrogenase (XDH), which catalyzes the two-step oxidation of hypoxanthine via xanthine to uric acid. During this oxidation NAD^+^ is used as electron acceptor. In contrast to XDH, XO prefers to use O_2_ as an electron acceptor, thus generating ROS, such as O_2_^•-^ or H_2_O_2_. Conversion of XDH into its XO form can occur either reversibly, due to disulfide bond formation or irreversibly via proteolysis [82,83] Different ROS-producing oxidases and ROS scavenger enzymes in peroxisomes were summarized by Schrader and Fahimi [65].

The ER has several mechanisms contributing to cellular ROS formation. In contrast to the cytosol, the ER represents a more oxidative environment, mainly due to the lower glutathione (GSH)/glutathione disulfide (GSSG) ratio compared to that of the cytosol (ER: 1:1–3:1; Cytosol:3000:1) [84,85]. This enables oxidative protein folding in the ER, generating intramolecular disulfide bonds to stabilize protein structure. The oxidation of two sulfhydryl groups is catalyzed by protein disulfide isomerase and the ER protein Ero1p, including several disulfide exchange reactions between these proteins. In the process, protein disulfide isomerase is reduced and then oxidized again by Ero1p. In the subsequent oxidation of Erop1 electrons are transferred to O_2_, whereby H_2_O_2_ is formed [69,70]. The desaturation of fatty acids in the ER requires an enzyme system containing NADH cytochrome b5 reductase and cytochrome b5, which act as a transfer component of electrons. This enzyme system represents another source of ROS, because of a potential electron transfer from NADH cytochrome b5 reductase to oxygen [71]. Examples for membrane-associated enzymes and enzyme systems capable of producing ROS are the microsomal monooxygenase (MMO) system or nicotinamide adenine dinucleotide phosphate (NADPH) oxidase (Nox). The MMO system occurs in the membrane of the ER and is involved in the metabolism of xenobiotics. The ability of the MMO system to produce O_2_^•-^ and H_2_O_2_ is known for several decades [72]. The complex reasons for the release of ROS, mainly based on an electron leakage in an electron transport chain, are described in detail elsewhere [86].

Another major cellular source for ROS is the membrane-associated enzyme complex Nox. Nox enzymes are generally inactive and divided into membrane-bound and cytosolic complexes, whereby the latter translocate to the membrane after induction, e.g., by the presence of bacteria, leading to production of bactericidal ROS. O_2_^•-^ is generated by an electron transport chain transferring electrons from the donor NADPH to molecular oxygen. Seven members of the Nox family are known, which are located in various cells and tissues and are induced in different ways [67,68].

In cellular redox metabolism, several redox equivalents or couples are involved in the exchange of electrons. Redox couples such NAD^+^/NADH, NADP^+^/NADPH and 2 GSH/GSSG are essential for the regulation of many cellular processes. Their oxidized to reduced ratios and concentrations reflect the cellular redox state, thus affecting the generation of ROS.

Both, NAD^+^ and NADP^+^, are dinucleotides, functioning as redox carriers in countless biochemical pathways. The two molecules can act as cofactors in enzymes or as substrates for redox reactions [87]. Furthermore the two redox couples differ in the respective ratio of oxidized to reduced molecules. In rat livers, the NAD^+^/NADH and NADP^+^/NADPH ratios were determined. The mitochondrial membrane is impermeable for NAD^+^, NADH, NADP^+^ or NADPH, resulting in separate cellular pools of these molecules and thereby also distinct oxidized/reduced ratios [88]. In the cytoplasm, the range of NAD^+^/NADH is 700-1,000, whereas in the mitochondria the ratio is 7–8. In contrast, the cytoplasmic NADP^+^/NADPH ratio is 0.01 [89,90]. Hence, mainly reductive processes are favored by NADPH [91]. Prominent examples for NADPH-dependent enzymes are the members of the Nox family, which are generating ROS due to the reduction of oxygen [67]. A further NAD^+^ pool is located in the peroxisomes, generated by a peroxisomal membrane-bound transporter for NAD^+^ and other cofactors [92]. Cellular NAD^+^ concentrations differ between tissues and the cellular pools have diverse NAD^+^ contents [91].

GSH is an atypical tripeptide consisting of the amino acids *L-γ*-glutamic acid, *l*-cysteine and glycine. GSH serves several cellular functions, most of which involve the thiol at its cysteine sidechain. On the one hand, GSH can be used for phase II metabolization of xenobiotics, where the nucleophilic cysteine group of GSH reacts with molecules activated in phase I. On the other hand, the cysteine group has redox-active properties, serving as a scavenger of ROS, such as H_2_O_2_. The reduction of ROS and the resulting oxidation of GSH leads to the formation of the disulfide GSSG, catalyzed by glutathione peroxidase [93]. The enzyme GSSG reductase reduces the accruing GSSG back to GSH using NADPH as electron donor [94,95]. Production of GSH takes place in the cytosol, depending on the availability of cysteine and the activity of the enzyme glutamate cysteine synthetase (GCS). The intracellular concentration of GSH may reach up to 15 mM [96], mainly distributed between cytosol, mitochondria and ER [84,97]. As mentioned above, GSH occurs predominantly in the reduced state [84], thus protecting thiols from irreversible oxidation by oxidants, such as ROS. Additional to the reduction of reversibly oxidized thiol groups, GSH can also prevent irreversible oxidation of thiol groups by reversible oxidative binding to cysteine residues of proteins, called glutathionylation [98]. In the following sections the chemical properties of thiols in metallothionein (MT) and other proteins as well as their role in cellular redox metabolism, particularly regarding the trace element zinc and its homeostasis, is described in more detail.

## 3 Zinc physiology

The human body contains 2–3 g zinc, making it the second most abundant trace element after iron [99,100]. It is estimated that zinc is a co-factor of 3,000 proteins, including more than 300 enzymes [101]. The largest amount of zinc is found in the muscle (49.5%). In descending order follow bones (36.7%), liver and skin (7.6%), and other tissues (6.2%) [102]. After resorption in the small intestine, the distribution of zinc to the different tissues is mediated via the plasma, where zinc is bound to proteins, mainly to serum albumin and α 2-macroglobulin [103,104]. The homeostasis of zinc is regulated through two classes of zinc transport proteins. On the one hand, the transporter family Zrt-, Irt-like protein (ZIP), which increases the cytosolic zinc concentration, and on the other the “zinc transporter” family (ZnT), which decrease cytosolic zinc concentration through the transport of zinc into cellular compartments or the extracellular space. Fourteen transporters belong to the ZIP family (ZIP 1–14 (SLC39A1-14)) and ten transporters are members of the ZnT family (ZnT 1–10 (SLC30A1-10)) [105], reviewed in detail elsewhere [106,107]. The total cellular zinc concentration is in the range of several hundred micromolar [108]. This intracellular zinc is distributed between cytoplasm (~ 50%), nucleus (~ 30–40%), and the membrane (remainder) [109,110]. The vast majority of cellular zinc ions are bound to proteins, which are either essential for cellular zinc homeostasis, such as MT, or require zinc to maintain their structure or catalytic activity.

Protein-bound zinc has a myriad of important functions. Since the first “zinc-finger” structure was discovered in the 1980's it is known that zinc has a structural role in transcription factors [111,112]. In the following decades the number of known zinc finger proteins increased and it was indicated that zinc finger proteins are a significant part of the transcriptional activators of the human genome [113]. Besides the structural zinc sites in proteins, three other main types of zinc-binding sites exist, catalytic, co-catalytic, and protein interface sites [114,115]. Mainly four amino acids coordinate zinc in proteins; cysteine (cys), aspartic acid (asp), histidine (his) and glutamic acid (glu). Depending on the zinc binding site in the individual protein, zinc is going to be preferably coordinated by different amino acids. Whereas cys-zinc coordination occurs predominantly in structural zinc sites, glu- and asp-zinc coordination may be found in catalytic zinc sites. The amino acid his can coordinate zinc in both zinc binding sites [116].

The fraction of zinc in the cell that is loosely bound to proteins or easily replaceable ligands is called free or labile zinc [117]. The concentration of free zinc varies, depending on cell type and the approach used for its measurement, between picomolar and low nanomolar [118]. Permanent excess or shortage of free zinc are detrimental, because they can lead to inhibition by zinc or loss of protein structure, respectively, and thus to protein dysfunction [118,119]. In case of a temporary increase of the free zinc concentration to 10^-8^M [118], called “zinc wave” or “zinc flux” (lasting seconds to minutes) or homeostatic zinc signals (persisting for hours), zinc acts as a second messenger [[120], [121], [122]]. Free zinc fluctuations are caused in response of the cell to external stimuli, such as lipopolysaccharide (LPS) or Phorbol-12-myristate-13-acetate [123,124]. Furthermore, the role of zinc as a second messenger in the signal transduction in monocytes and macrophages after stimulation with LPS was shown [124,125]. Three general pathways exist to generate an increase of extracellular or intracellular free zinc concentrations due to a release of zinc ions [126]. Redox signals represent one of these mechanisms for an increase of the cytosolic free zinc concentration. They can lead to oxidation of the zinc binding amino acid side chains, specifically cysteine thiols, resulting in an intracellular oxidative release of zinc ions from proteins [18,[127], [128], [129]]. The other two pathways include the release of zinc ions via vesicular exocytosis and via channels either importing/exporting extracellular zinc or sequestering zinc from intracellular stores, leading to extracellular or intracellular increased free zinc concentrations, respectively [126].

## 4 Interplay of zinc, thiols and MT

MT was first isolated in 1957 from equine kidney cortex. Back then, the unknown protein stood out because of its low molecular weight and high cadmium content [130]. Further investigations showed high contents not only of cadmium (2.9%), but also zinc (0.6%) and sulfur (4.1%). Due to these findings the protein was named MT [131]. Since its discovery, various functions and properties of MT were identified. Human MT can be divided into four classes (MT-1 to MT-4), comprising eleven functional isoforms eight of which belong to class 1 [132]. MT-1 and MT-2 are ubiquitously distributed in the human body and are expressed in many organs, whereas MT-3 is mainly present in the central nervous system and MT-4 in the skin and other stratified epithelium [133]. All isoforms have a molecular weight of approximately 7 kDa and lack aromatic amino acids [134]. In addition, they have twenty cysteine residues, which confer MT special properties based on the attributes of thiol groups [134,135].

Thiol groups have various biologically relevant features. They occur as nucleophiles in catalytic sites of enzymes or provide structural stability due to formation of disulfide bonds. Due to their redox properties, additional functions of thiol groups are scavenging of radicals, transmission of redox signals or complexation of metal ions, for instance zinc. Generally, the reactivity of the thiol group is important for cysteine functions, which depends, among other things, on its ionization state. A smaller pK_A_ value and thus easier ionization result in higher nucleophilicity, thus affecting their reactivity [136]. The cellular environment determines the ionization state and reactivity of the thiol group [137,138]. For instance, the vicinity of positively charged amino acids or hydrogen bonds facilitates ionization of cysteines and increases the reaction rate [[139], [140], [141]]. Hence pK_A_ values, reactivity, and thus the reaction rate, for instance with oxidants such as H_2_O_2_, differ considerably between thiol groups in individual molecules or enzymes [141].

Thiol groups can be oxidized and occur in different oxidation states (-SH, -SS-, -SO^-^, –SO_2_^-^, –SO_3_^-^) [135,142]. Oxidation of the thiolate group to sulfenic acid (RSO^-^) is an intermediate stage that may undergo further oxidations to sulfinic acid (RSO_2_^-^) and sulfonic acid (RSO_3_^-^) or react with another sulfhydryl/nitrogen group to form a disulfide/sulfenamide. The lower oxidation states (-SS-, -SO^-^) can be reduced back to sulfhydryl groups, for instance by the GSH/GSSG system [138,143]. In contrast, the reduction of RSO_2_^-^ and RSO_3_^-^ represents a challenge [135], whereby the reduction of RSO_2_^-^ by sulfiredoxins was shown [144,145]. Reversible thiol oxidation represents one of the most important reactions of cysteine residues *in vivo*. The electron transfer between two thiol modifications or one thiol modification and redox couples like NAD^+^/NADH is involved in processes such as oxidative protein folding, maintenance of the cellular redox potential or regulation of signaling and gene expression triggered by the exposome [146].

Due to the properties of its vast number of cysteine residues, MT is involved in the homeostasis of essential metals, for instance copper or zinc. Additionally, MT can also bind other metals for detoxification, such as cadmium, or can act as a redox buffer [147]. For both, copper and cadmium, MT has a higher affinity in comparison to zinc [147], but MT seems to be particularly important to maintain cellular zinc homeostasis and plays a critical role in its storage and redistribution [148]. The reduced, metal-free form of the protein, called apo-MT or thionein (T_red_), can bind up to seven zinc ions, four of them through complexation by eleven cysteines in the α-domain and three ions bound to nine cysteine residues in the β-domain [149]. In both of these zinc-thiolate clusters each zinc ion is coordinated by four thiolates. Overall the β-domain is more labile in comparison to the α-domain. Accordingly, it can be assumed that the β-domain is preferentially involved in physiological processes, such as scavenging ROS or homeostasis of essential metals, whereas the α-domain seems to be of higher relevance for the detoxification of heavy metals [149]. Accordingly, within one MT molecule, there exist three classes of zinc binding sites, which are distinguishable by their affinities [150]. Four zinc ions are strongly bound with high affinity, two zinc ions with intermediate affinity and one site binds the seventh zinc ion weakly with nanomolar affinity, leading to the hypothesis that a zinc-saturated MT does not occur under normal conditions *in vivo* [150,151]. Saturation of MT with zinc is possible, e.g., in case of a “zinc flux”, when the intracellular free zinc concentration increases to nanomolar, matching the affinity of the weak zinc binding site [150,152].

Increased intracellular free zinc concentrations induce expression of T_red_ via the metal regulatory transcription factor 1 (MTF-1), resulting in zinc-sequestration by T_red_ and thus a downregulation of its availability [153,154]. Next to zinc-buffering, it is suggested that MT is also involved in a specific part of regulation of zinc homeostasis, called zinc-muffling [120]. Zinc muffling includes intracellular processes that change cytosolic zinc concentrations “under non steady state conditions”, whereas zinc-buffering includes processes “under steady state conditions”, as introduced by Colvin et al. [155] Whereas thermodynamic zinc-buffering includes binding and release of zinc in the cytoplasm in order to maintain the free zinc concentration in a picomolar range, zinc-muffling is a kinetic process and contains, for instance, sequestration of zinc either outside the cell or into cellular organelles. The role of MT in zinc-muffling comprises the transfer of zinc to its transporters, which are localized at the plasma membrane or membranes of the respective organelles [120,155]. Hence, T_red_ and MT are an essential part of intracellular zinc homeostasis, tightly controlling the intracellular free zinc concentration.

**Fig. 1:**
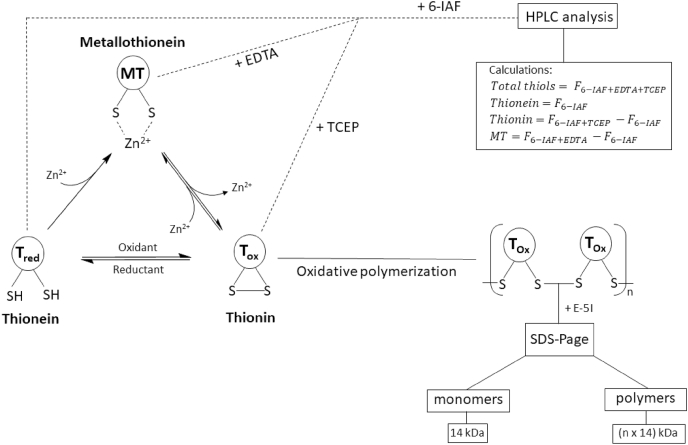
MT species occurrence and detection. The cysteine residues of the apo-protein thionein (T_red_) can complex up to seven zinc ions, generating the holo-protein metallothionein (MT). By reactions with cellular oxidants, such as ROS or disulfides, the thiol groups are oxidized to the oxidized form thionin (T_ox_). Thiol oxidation generates intramolecular or intermolecular disulfide bonds, with concomitant release of zinc, whereas the reduction to T_red_ induces restoration of zinc-binding. The formation of intermolecular disulfide bonds leads to MT polymers. All three forms of MT can be simultaneously present in the cell. Two assays were developed for analyzing the different MT species by characterizing metal content and oxidation state. One assay is based on sodium dodecyl sulfate polyacrylamide gel electrophoresis (SDS-PAGE) after thiol labeling for the investigation of polymer formation due to size separation. Each monomer in combination with the derivation agent has an apparent molecular weight of 14 kDa. Alternatively, the quantification of the different species can be investigated with high performance liquid chromatography (HPLC) after labeling of the free thiol groups with the fluorescent dye 6-iodoacetamidofluoresceine (6-IAF). The amounts of each species can be calculated as the difference between four fluorescence measurements (F). The fluorescence without adding any agents corresponds to the amount of T_red_. After addition of a chelating (ethylenediaminetetraacetic acid (EDTA)) or a reducing (tris(2-carboxyethyl)phosphine (TCEP)) agent the fluorescence increases by the amount of metal-bound or oxidized thiol groups, respectively. The difference between the fluorescence after adding chelating or reducing agents and the fluorescence without adding both agents corresponds to the respective amounts of MT or T_ox,_ while measurement in the presence of both TCEP and EDTA gives the sum of all species [19,151,156]..

In particular during the process of zinc-buffering by MT the redox properties of the thiol groups play a critical role. The cysteine residues in T or MT can be oxidized by cellular oxidants, for example ROS or GSSG, leading to the formation of intramolecular or intermolecular disulfide bonds, resulting in the generation of thionin (T_ox_) or disulfide-linked MT polymers [19,151,156,157]. In the case of MT the oxidation of the thiol groups results in a release of zinc ions and generates a “zinc flux” [19]. Vice versa, by reducing T_ox_ to T_red_, the protein can regain its zinc-binding capability (Fig. 1) [158]. In addition, it was demonstrated that MT is able to transfer zinc to other proteins [159].

All three forms of MT co-exist in the cell [156,160]. In general, MT is localized in the cytoplasm and can translocate into the nucleus, to protect DNA from damage and interact with transcription factors [161]. The interaction and scavenging of RS and other oxidants suggests that MT is also a redox buffer [162]. Thiol oxidation by RS releases zinc ions [19], which can induce zinc signals and hereby also the expression of additional T_red_ to scavenge further RS [153]. The expression of T_red_ is not only activated by zinc. Aside of other metal ions, such as cadmium, also RS, LPS or other molecules that trigger oxidative stress can upregulate expression of T_red_ [153]. Among the entirety of MT-inducing metals, xenobiotics and RS, MT expression is most prominently stimulated by cadmium [153,154]. MT expression can be induced by multiple response elements in the MT promoter region: the metal response element (MRE) [154], the antioxidant response element (ARE) [163] and the glucocorticoid response element (GRE) [164]. In the case of zinc, the expression of T_red_ is activated via the transcription factor MTF-1. MTF-1 is a zinc finger protein and contains six zinc finger domains [165]. After zinc binding, MTF-1 translocates into the nucleus and binds to the MRE promoter region of target genes, hereby upregulating the transcription of T_red_ and other proteins, such as the zinc transporter ZnT 1 or the enzyme GCS [154,166]. Therefore, MTF-1 is a zinc sensor and induces the expression of various antioxidants and other proteins in a zinc-dependent manner [166]. Cadmium and ROS or other oxidants can also induce expression of T_red_ [154]. In contrast to zinc, H_2_O_2_- or cadmium-mediated T_red_ expression occurs via a combination of two response elements MRE and ARE [154]. It was shown that the ARE region is responsive to H_2_O_2_ and cadmium, but not zinc. In *in vitro* studies in murine cells, deleting the ARE promoter region of the gene for MT-1 attenuated T_red_ expression in response to H_2_O_2_ and cadmium [154]. In addition, indirect H_2_O_2_- or cadmium-mediated T_red_ expression via MRE is based on zinc release from zinc proteins, resulting in an increase of free zinc and the zinc-dependent activation of MTF-1 [154,167].

The lack of aromatic amino acids in MT represents a challenge for quantitative MT analysis, because standard methods, such as measuring the absorption at 280 nm, are not applicable [134]. Investigation of MT speciation with regard to metal content or oxidation state, is at least equally challenging. Yang et al. developed an assay for simultaneous quantification of the apo-protein T_red_ and the holo-protein MT [160]. Additionally, an updated method also suited for determining T_ox_ was described (Fig. 1) [156]. This assay is based on labeling of free thiol groups with the fluorescent dye (6-iodoacetamidofluoresceine) 6-IAF. After adding a chelating or a reducing agent, the respective metal-bound or oxidized thiols become accessible for reacting with 6-IAF, allowing a calculation of the relative fractions of the different thiol species by subtracting the signals of free thiols from those obtained in the presence of reducing agents, chelators, or both (Fig. 1). As mentioned above, MT can polymerize through formation of intermolecular disulfide bonds [157]. The polymerization can be analyzed by detecting proteins with molecular weights of multiples of 14 kDa, corresponding to the apparent molecular weight of a monomer in combination with the derivatization agent (Fig. 1) [151].

## 5 Zinc and redox signaling

The term redox signaling has no uniform definition in the literature [[168], [169], [170], [171]]. For example, Forman et al. characterized redox signaling as involving either a redox reaction or a “covalent adduct formation between the sensor signaling protein and second messenger” [171]. With regard to quantitative measurements of redox signaling, Pillay et al. proposed a working definition for redox signaling: “a kinetic process involving coordinated changes in the oxidation state of redox transduction machinery leading to specific outputs in response to hydrogen peroxide and other oxidants” [169]. Overall, important points for redox signaling processes are the participation of RS and the specificity of the induced reactions and pathways. In contrast to conventional cell signaling pathways, which include second messengers such as cyclic adenosine monophosphate or cyclic guanosine monophosphate, redox signaling involves second messengers with a high potential to undergo unspecific reactions [171]. The wide range of potential reactions of RS comprises, in addition to the reactions that take part in redox signaling, also non-specific oxidations of macromolecules without further signal transduction [169,171]. The reaction of RS with specific targets in redox signaling frequently includes the oxidation/reduction of cysteine residues, which play an essential role in redox metabolism and signaling. The different reactivity and nucleophilicity of the targets, which are determined by their ionization state or their protein environment as already outlined above, provides one part of redox signaling specificity. The other part of this specificity is cellular localization. A short distance between the electrophile and the target protein increases specificity [171]. The major second messengers in redox signaling are peroxides, such as H_2_O_2_. Other ROS with higher reactivity, such as O_2_^•-^ or OH^•^ lack specificity [171]. Currently, several mechanisms are known by which H_2_O_2_ participates in physiological signaling, such as the regulation of transcription factor activity [172,173].

**Fig. 2:**
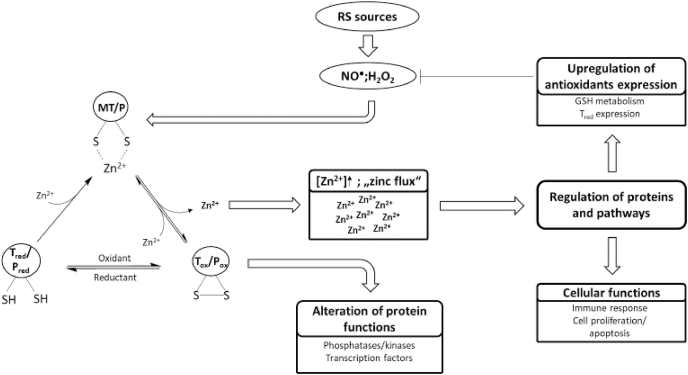
Redox signaling and its transformation into zinc signals. Redox signals in the form of RS or other cellular oxidants can oxidize cysteine residues in proteins, resulting in the oxidative release of thiol-bound zinc ions. The proteins that are oxidized can be redox transducers or redox sensors. The redox transducer translates the redox signal into a zinc signal, which can influence the activity of other proteins and can regulate different cellular functions or pathways, such as cell proliferation or expression of antioxidative molecules, respectively. Thus, a redox transducer is oxidized to affect the functions of other proteins. In case of a redox sensors, the aim of the redox signal is the change of the target protein function due to its oxidation, while eventually released zinc is not involved in signaling.

Zinc has a dual role in redox signaling. On the one hand, it attenuates oxidation of cysteine residues, whose reactivity is diminished upon binding of zinc due to sterical hindrance and its action as a Lewis acid. On the other hand, zinc can be a second messenger in the redox-dependent signaling cascade, subsequent to oxidative release from zinc thiolate complexes, for instance those in MT (Fig. 2) [19].

The target proteins of this reversible oxidation, also called redox zinc switches [174], can be divided into redox sensors and redox transducers. Redox sensors are oxidized to change their own function, whereas redox transducers are oxidized to affect other proteins or pathways. A redox sensor can therefore be considered as the recipient of a specific redox signal, whereas the redox transducer is a part of the redox signaling cascade. While zinc that may be released during oxidation of a redox sensor has no specific function, zinc ions that are released from redox transducers actually comprise the next stage in the signaling cascade [175]. Hereby, the redox transducer translates a redox signal into a zinc signal [18,175]. An excellent example for a redox transducer is MT, whereby MT is suggested as “a link between redox and zinc signaling” [175] connecting free zinc and the cellular redox state (Fig. 2) [176].

Increase of free zinc can induce several pathways or transcription factors to regulate antioxidative defense or cellular functions, such as proliferation or apoptosis (Fig. 2). Selected transcription factors or pathways that are influenced by zinc or redox signals are discussed below.

Besides MTF-1, another zinc-sensitive transcription factor is the nuclear factor erythroid 2-related factor 2 (Nrf2). Nrf2 is also a zinc finger protein and translocates into the nucleus after activation [17]. Whereas MTF-1 binds to the MRE promoter region, Nrf2 binds to the ARE promoter region and activates the transcription of multiple antioxidative proteins in response to various kinds of stress [177,178]. Under normal conditions Nrf2 is bound to its repressor protein Kelch-like ECH-associated protein 1 (Keap1), which promotes the degradation of Nrf2. An oxidative modification of cysteine residues from Keap1 leads to conformational changes and the stabilization of Nrf2 [179]. For instance, Nrf2 regulates GCS expression and thus is essential for the regulation of GSH metabolism [180]. Next to oxidants, zinc can also change the conformation of Keap1 and prevent the degradation of Nrf2 [181]. Several *in vitro* and *in vivo* studies support the hypothesis of zinc regulating the activity of Nrf2, thus mediating the expression of antioxidative proteins [[182], [183], [184]]. In addition, there are a number of other zinc-modulated transcription factors or zinc proteins (thereby being potential targets for zinc released from redox transducers), such as nuclear factor κB (NF-κB), tristetraprolin, the kruppel-associated box domain proteins or the hepatocyte nuclear factor-4α, which were recently reviewed by Prasad and Bao [17].

Protein phosphatases are a subgroup of phosphatases that dephosphorylate amino acid side chains in proteins. Depending on the specificity for the dephosphorylated amino acid, protein phosphatases can be differentiated into protein tyrosine phosphatases (PTPs), protein serine/threonine phosphatases (PSPs) or dual-specificity phosphatases (DUSPs), which can dephosphorylate all three amino acids [185]. The mechanisms of phosphorylation and dephosphorylation of proteins are essential for cellular signal transduction. Hence kinases and phosphatases are regulating cellular signaling and alterations in their activity are connected to a variety of diseases, such as cancer or autoimmune diseases [186,187].

PTPs can be inhibited by zinc at micromolar, nanomolar, and sometimes even picomolar concentrations [188]. This inhibition is reversible by chelating agents [189], such as ethylenediaminetetraacetic acid (EDTA), or cellular molecules, such as T_red_ [190]. PTP 1B plays an essential role in insulin signaling by dephosphorylating the insulin receptor [191,192]. Zinc-inhibition of the PTPs 1B and SHP-1 (IC_50_ values: 17 and 93 nM, respectively) in C6 rat glioma cells resulted in increased phosphorylation of the insulin receptor and the insulin receptor substrate, without affecting activity of the corresponding kinases [193]. Moreover, inhibition of the human T-cell PTP with an IC_50_ value of 200 nM was observed [190], and the receptor protein-tyrosine phosphatase β was inhibited by picomolar concentrations of zinc [194]. In addition, inhibition of the phosphatase and tensin homolog deleted on chromosome 10 (PTEN) by zinc occurs at approximately 0.6 nM [195]. PTEN is a tumor suppressor and dephosphorylates the second messenger phosphatidylinositol (3,4,5)-trisphosphate, which activates the kinase Akt. Hence, inhibition of PTEN by zinc leads to the activation of cell proliferation and antiapoptotic processes [195]. Physiological zinc concentrations that occur in case of a “zinc flux“ are in the range of the zinc concentrations capable of inhibiting PTPs, suggesting that these are a relevant target of zinc signals [188,196].

Next to inhibition by zinc, two further ways exist to influence the activity of PTPs, which have both been reported for PTP 1B [197,198]. One is a modulation of the activity by phosphorylation [198]. The other, in the context of this review more relevant one, is the reversible oxidation of the cysteine in the catalytic site, e.g., of PTP B1, resulting in its inactivation [197]. The catalytic cysteine of PTP B1 has a low pKa value (~3.8–5.6) and is thus prone for oxidation [199]. PTPs can be inhibited, e.g., through oxidation with H_2_O_2_, and reactivated by thiols, such GSH [200]. As another example, the reversible oxidation of PTEN after cell stimulation with growth factors was reported [201]. In fact, insulin and other growth factors induce not only phosphorylation cascades, but also the generation of H_2_O_2_, resulting in the oxidative inhibition of phosphatases such as PTP 1B or PTEN [197,201]. In various PTPs the oxidation of the catalytic cysteine initially leads to different oxidation states of sulfur (disulfides, sulfenic acid (RSO^-^), sulfinic acid (RSO_2_^-^) or sulfonic acid (RSO_3_^-^)) [202]. Subsequent reactions with neighboring amino acid residues can result in intramolecular crosslinking, due to a sulfenylamide structure or disulfide bonds and thus to structural and conformational changes [203,204]. Notably, it has been shown that zinc binding does not just inhibit PTEN activity, but oxidation of the cysteine residues Cys 124 and Cys 71 of PTEN can be prevented when zinc is bound to the phosphatase. Therefore, temporary reversible inhibition of PTEN, and potentially other PTPs as well, by zinc interferes with their redox-regulation, averting permanent oxidative inhibition [195]. In addition to cysteines, other amino acid residues in phosphatases might be oxidized as well, leading to a loss of enzymatic activity due to alterations in protein folding. Oxidation of the phosphatase PTEN with HOCl results in oxidative modifications such as chlorination or hydroxylation of tyrosine, tryptophan or proline [205]. Yet, to date no effect of zinc on these events has been reported.

Regulation of DUSPs by zinc [206] or oxidation [207] has been shown for mitogen-activated protein kinase phosphatases (MAKPs), which attenuate signaling cascades involved in cell proliferation by dephosphorylation of kinases. The zinc regulation of DUSPs has been shown by a zinc-dependent phosphorylation of the protein kinases mitogen activated protein kinase kinase (MEK) and extracellular signal-regulated kinases (ERK) after interleukin-2 stimulation of T cells, due to inhibition of MAKPs [206]. Furthermore, during oxidative stress in neurons, cysteine thiol oxidation leads to reversible inhibition of ERK-directed phosphatases [207].

For the third group of protein phosphatases, PSPs, Denu and Tanner observed no oxidative inhibition of selected phosphatases by H_2_O_2_^200^, whereas Santos et al. demonstrated inactivation of the PSP protein phosphatase 1 by H_2_O_2_ [208]. Addition of reducing thiols did not reactivate protein phosphatase 1, leading to a suggested mechanism involving the oxidation of the dinuclear metal center, in contrast to the catalytic thiol residues that are affected in PTPs [208]. Additionally, the PSPs protein phosphatase 2A and λ Ser/Thr phosphoprotein phosphatase are inhibited by zinc *in vitro* [209,210].

The results summarized above identify protein phosphatases, in particular PTPs, as an interface between redox and zinc signaling, regulated by a triad of relevant mechanisms. First, they are redox sensors. Second, they are also controlled by zinc signals that can be generated due to oxidation of redox transducers. This makes phosphatases molecules where signals from redox transducer-generated zinc fluxes and redox sensors converge. Third, zinc binding protects cysteine residues in phosphatases from oxidation, making phosphatases a place for crosstalk between zinc- and redox signaling.

In signal transduction, protein phosphatases are the antagonists of protein kinases. Together they regulate the activity of proteins by the transfer of phosphate groups. Hence, protein kinases are, similar to phosphatases, involved in cellular signaling processes regulating apoptosis or cell proliferation. The activity of several kinases is influenced by zinc or redox signals. MAPK, comprising ERK, Jun N-terminal kinases (JNK) and p38 as their main subgroups [211] are involved in signaling pathways regulating cell proliferation and cell death. They are activated through stress stimuli (JNK, p38) or growth factors (ERK). Hence, ROS can induce stress that, in turn, activates the MAPKs JNK and p38 [212]. For example, the apoptotic signal-regulating kinase 1 (ASK1) is involved in p38 and JNK cascades and is responsive to ROS. Its inactive form is bound to reduced thioredoxin, which inhibits the activity of ASK1 [213]. Upon the oxidation of the cysteine residues Cys 32 and Cys 35 in the redox active site of thioredoxin, ASK1 dissociates. Autophosphorylation of a threonine residue (Thr838) by ASK1 then leads to reactivation of its kinase activity, initiating downstream signaling [214]. It seems possible that the critical cysteine residues in thioredoxin bind zinc, which either protects cysteine residues against weaker oxidants or in case of stronger oxidants is released and plays a role as a second messenger. While this has not yet been shown in mammalian signal transduction, it was already observed in *E. coli* that H_2_O_2_ was able to release zinc from thioredoxin 2 [215]. In addition, indirect activation of ERK by ROS was also shown to result from activation of growth factor receptors [216].

Zinc has a differential influence on the individual groups of MAPKs. For example, only p38 is selectively activated in human T-cells by incubation with zinc (in contrast to ERK1/2 or JNK), while after stimulation of the T-cell receptor the phosphorylation of ERK1/2 was inhibited by zinc in a concentration-dependent manner [211]. This can be based on different molecular effects, e.g., zinc-inhibition of specific MAPK-phosphatases can result in higher phosphorylation of ERK1/2 and, alternatively, zinc may also affect receptors upstream of ERK [217], such as PDE/cyclic nucleotide/PKA-mediated inhibitory phosphorylation of Raf-1, which acts upstream of ERK1/2 [218]. The impact of zinc on ERK1/2 activation or phosphorylation state is well documented and modulation of ERK and other MAPK activities has been comprehensively reviewed elsewhere [219,220].

Another important kind of kinases is the protein kinase C (PKC) family, a group of serine/threonine kinases. The catalytic and regulatory domains of PKCs contain cysteine residues that can be oxidized by cellular oxidants. In the regulatory domain C1, two zinc finger domains are present and coordinate zinc ions [221]. In case of an oxidative zinc release due to oxidation of the regulatory domain the activity of PKC increases [222]. In contrast, oxidation of thiol groups in the catalytic domain results in inactivation of PKC [221]. The oxidative inhibition due to oxidation of the catalytic domain requires higher concentrations of oxidants [221]. Hence, conditions of oxidative stress can modulate PKC activity depending on the amount of ROS, affecting several downstream signaling pathways. In addition, Slepchenko et al. showed a regulation of the function of PKC *delta* by zinc. They suggest a phosphorylation on threonine residue 505 (Thr^505^) in a free zinc concentration-dependent manner. In case of high intracellular zinc concentrations, a zinc-binding domain blocks the phosphorylation at Thr^505^ [223].

The caspases, which include 13 members, are cysteine endopeptidases and cleave proteins after aspartic acid sites [224]. Zinc inhibits several caspases. For instance, its half maximal inhibitory concentration for caspase-3, a central effector caspase in apoptosis, was initially found to be <10 nM [190]. In addition, for the caspases 3 (6.9 nM), 6 (2.6 nM), 7 (76.1 nM), and 8 (4.3 nM) nanomolar inhibiting constants were later observed, indicating that they could be relevant targets for zinc *in vivo* [225]. Caspase 8 binds two zinc ions, one of which is located in the active site of the enzyme (Cys360), while the other zinc binding site remains unknown. Zinc binding to caspase 8 prevents formation of its active form, which is a caspase 8 dimer [225]. Caspases 3 and 7 have three and one zinc binding sites, respectively. In both caspases, one inhibitory zinc binding site is located in the active catalytic dyads of the enzymes [225,226]. In contrast, the inhibitory zinc binding site of caspase 6 is allosteric, involving the amino acids Lys-36, Glu-244 and His-287 [227]. Zinc inhibition of caspase 9 was reported in the micromolar range [228,229]. Huber et al. investigated two binding sites in caspase 9 for zinc inhibition. One site involves the catalytic site of caspase 9, in particular the catalytic histidine (His 237) and cysteine (Cys 287) residues, whereas the other site comprises a cysteine residue (Cys 272) that is located away from the catalytic site [229]. Aside of their inhibition by zinc, caspases can also be regulated by oxidative modifications, such as glutathionylation [230], nitrosylation [231] or persulfidation [232]. Caspase 3 was shown to be inhibited by micromolar concentrations of GSSG (IC_50_: 75 μM), due to glutathionylation of the cysteine residues Cys 45 on p12 subunit and Cys 135 on p17 subunit (active site), whereby the inhibition is reversible by the addition of reducing agents, such as GSH [230]. Consequently, caspase 3 can already be oxidatively inhibited by physiological GSSG concentrations (up to 120 μM) [233]. Nitrosylation was reported to inhibit the active cysteine residue of caspase 3 as well as to decline the cleavage of procaspase 9 into its active form and consequently decreases apoptosis [231,234].

It seems worthwhile to look for parallels in the regulation of caspases and PTPs. Both enzyme families have an active site cysteine, and several members of the respective families have been shown to be a target for regulation by zinc as well as redox. While this has not yet been substantiated by experimental evidence, the active site cysteine in caspases might be a target for multiple forms of crosstalk between zinc and redox signaling similar to the one in PTPs, including a protective effect of zinc from oxidative inactivation.

## 6 Alterations in zinc status and their influence on redox homeostasis and signaling

Zinc deficiency can occur due to several reasons, including malnutrition or other dietary causes, such as the intake of high amounts of zinc-chelating factors, most prominently phytate [235]. This nutritional deficiency is linked to increased apoptosis, chronic inflammation and oxidative stress, potentially contributing to chronic diseases [17,236,237]. Especially elderly people are prone to zinc deficiency, resulting in impaired efficiency of the immune system and other systematic dysfunctions [238,239]. These adverse effects of zinc deficiency in the elderly can be reversed by zinc supplementation [240]. Zinc excess due to disproportionate zinc supplementation can also lead to oxidative stress [18,241]. In general, high zinc levels can interrupt important processes, such as citrate acid cycle or glycolysis, due to a reduction in NADH production [242] or enzyme inhibition of phosphofructokinase [243]. Furthermore, zinc activation of lipoamide dehydrogenase, which is a part of the α-KDH complex and catalyzes the production of H_2_O_2_, leads to an increase of ROS production [244]. Overall, zinc has predominantly antioxidative functions, whereas alterations in zinc status lead to prooxidative conditions [18].

Furthermore, on the cellular level zinc deficiency or excess influence redox homeostasis and redox signaling. Alterations in zinc status can lead to an overproduction of RS and thus to oxidative stress. Accordingly, the activity of antioxidative enzymes, such as copper/zinc SOD is increased during zinc deprivation, acting as a protective mechanism against oxidative damage [245]. Vice versa, copper/zinc SOD activity is reduced during zinc excess, because copper is essential for the activity of copper/zinc SOD and elevated zinc levels can lead to copper deficiency [18,246]. Normal zinc levels are essential for expression of antioxidative molecules, resulting in a significant shift of the pro-oxidative/anti-oxidative balance to the side of pro-oxidative conditions during zinc deficiency. These oxidative conditions affect various cellular pathways or transcription factors, which are involved in mechanisms such as cell proliferation or apoptosis [245]. For instance, zinc deficiency reduced the tyrosine phosphorylation of the transcription factors signal transducer and activator of transcription (STAT) 1 and STAT 3, whereas serine phosphorylation is increased, due to the activation of the MAPK p38 by oxidative stress [247]. In addition, oxidative conditions during zinc deficiency impair the translocation of STAT 1 and STAT 3 into the nucleus. Furthermore the MAPKs p38 and JNK activity is increased during zinc deficiency, whereas ERK 1/2 activity is decreased*.* [247,248] In addition, the binding activities of other transcription factors, such the activator protein 1 (AP-1) or NF-κB, are increased or decreased, respectively, by zinc deficiency. This is in accordance with the theory of higher ROS levels or lower amounts of antioxidants during zinc deficiency, as dysregulation of redox equilibrium modulates these transcription factors [245,248,249].

An example for the disrupted generation of antioxidative molecules during zinc deficiency is the reduced formation of GSH in *in vitro* studies, due to inhibited expression of the glutamate cysteine ligase subunits, but also through the activation of Caspase 3, which cleaves the GCS, an essential enzyme for GSH generation [250].

## 7 Conclusion

Zinc plays an essential role in redox metabolism and signaling. Alterations in cellular zinc homeostasis are crucial for the prooxidative/antioxidative equilibrium and can lead to a shift to prooxidative conditions, resulting in life threatening diseases. Zinc and redox signals are connected by the oxidative release of zinc from proteins, for example MT, due to oxidation of thiol groups, switching a redox signal into a zinc signal. This increased free zinc concentration, called “zinc flux”, affects proteins such as phosphatases or caspases and pathways regulating cell proliferation, apoptosis or expression of proteins, e.g. T_red_. In addition, zinc can protect proteins from oxidation thereby preserving their functions.

However, Maret suggested that these investigations need to be carefully reinterpreted to distinguish physiological zinc signaling from pathophysiological influences on protein functions, considering the physiologically occurring free zinc concentrations [18]. Another point that needs to be questioned is the representativeness of *in vitro* cell culture tests for investigations regarding redox metabolism and signaling. The higher amounts of ROS in cell culture experiments may lead to results that do not adequately represent redox-related processes *in vivo*.

Taken together many parallel targets and interactions for zinc and redox metabolism have been described. Yet, the physiological relevance of existing observations as well as a myriad of unidentified molecular sites of their mutual interaction remain to be elucidated.
